# Paraesophageal hernia with incarceration of the gastric antrum and duodenal bulb: a case report

**DOI:** 10.1186/1756-0500-6-451

**Published:** 2013-11-11

**Authors:** Nobuhiro Takeuchi, Yusuke Nomura

**Affiliations:** 1 Department of Gastroenterology, Kawasaki Hospital, 3-3-1, Higashiyama-cho, Kobe, Hyogo 652–0042, Japan

**Keywords:** Paraesophageal hernia, Incarceration, Surgery

## Abstract

**Background:**

In cases of esophageal hernia, incarceration of peritoneal organs other than the stomach is rare.

**Case presentation:**

An 84-year-old female was admitted to our institution with a complaint of nausea and vomiting. Abdominal computed tomography revealed an esophageal hiatal hernia with incarceration of the gastric antrum and duodenal bulb. Gastrofluorography under gastroendoscopy confirmed prolapse of the antrum and duodenal bulb into the esophageal hernial sac. Although gastroendoscopy guided repositioning of the prolapsed organs was successful, reprolapse occurred immediately. Therefore, surgical treatment was indicated. The gastric antrum and duodenal bulb were associated with a paraesophageal hernia. Therefore, they were repositioned, and passage from the duodenal bulb to the descending portion of the duodenum was improved.

**Conclusion:**

We report a rare case of paraesophageal hernia with incarceration of the gastric antrum and duodenal bulb.

## Background

In cases of esophageal hernia, incarceration of peritoneal organs other than the stomach is rare. We report a case of paraesophageal hernia with incarceration of the gastric antrum and duodenal bulb.

## Case presentation

**Figure 1:**
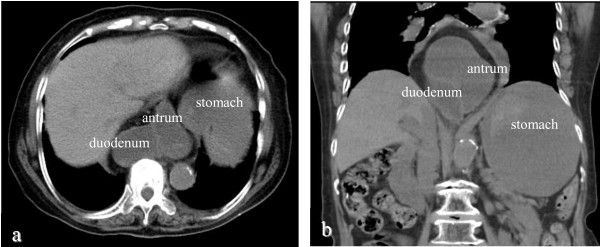
**Plain abdominal computed tomography.** The prolapse of the gastric antrum and duodenal bulb into the mediastinum were revealed. The stomach and duodenal bulb were markedly dilated and retention of fluid contents was evident (**a**: axial section, **b**: coronal section).

**Figure 2:**
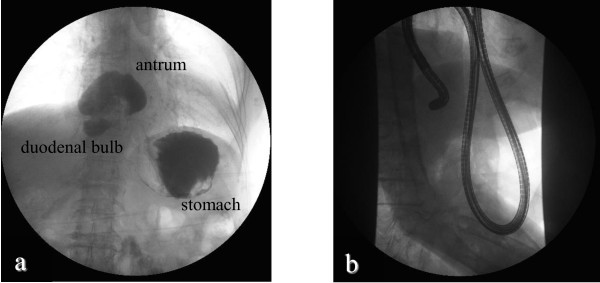
**Gastrofluorography.** The prolapse of the gastric antrum and duodenal bulb into the mediastinum were revealed **(a)** Prolapsed gastric antrum and duodenal bulb into the hernial sac were successfully repositioned under gastroendoscopic guidance and the passage of contrast medium from the duodenal bulb into the descending portion of the duodenum was improved **(b)**.

An 84-year-old female was admitted to our institution with a complaint of nausea and vomiting. Her medical history included cataract surgery, hypertension, and multiple lacunar infarctions. The patient had no history of smoking or alcohol intake. On admission, her blood pressure was 150/74 mmHg, heart rate was 86 beats/min, body temperature was 36.5°C, and oxygen saturation level was 98% in room air. On initial clinical examination, her weight was 44.5 kg, height was 142 cm and body mass index (BMI) was 22.1 kg/m^2^. Inspection of her palpebral conjunctiva revealed no evidence of anemia. Chest auscultation revealed no evidence of abnormal heart murmurs and no rales or other abnormal respiratory sounds. The abdomen was slightly distended, but normal peristalsis was evident. Mild tenderness was detected in her upper abdomen. No masses were palpable, and no signs of peritoneal irritation were detected. A physical examination revealed no edema or cyanosis. Although the chest radiography revealed a normal cardiothoracic ratio of 59.5%, the abdomen was markedly dilated and bloated, which was subsequently identified to be caused by the gastric antrum and duodenum incarcerated behind the heart. Abdominal computed tomography revealed an esophageal hiatal hernia with incarceration of the gastric antrum and duodenal bulb (Figure 1a and b). Blood chemistry analysis revealed an elevated white blood cell count (12,000/μL), normal C-reactive protein levels (0.1 mg/dL), an elevated glucose level (206 mg/dL), mild hypoproteinemia (6.5 g/dl), mild hypoalbuminemia (3.6 g/dl), and evidence of coagulation dysfunction (prothrombin time, 75%; international normalized ratio, 1.19). Upper gastroendoscopy revealed severe deformation of the stomach and the presence of massive contents within. Stenosis was evident in the region prior to the descending portion of the duodenum. Therefore, gastrofluorography under gastroendoscopic guidance was performed. Gastrofluorography confirmed the prolapse of the antrum and duodenal bulb into the esophageal hernial sac (Figure 2a and b). Although repositioning of the prolapse was successfully performed under gastroendoscopic guidance, reprolapse occurred immediately. Therefore, the following surgical treatment was performed on Day 11 of hospitalization.

**Figure 3:**
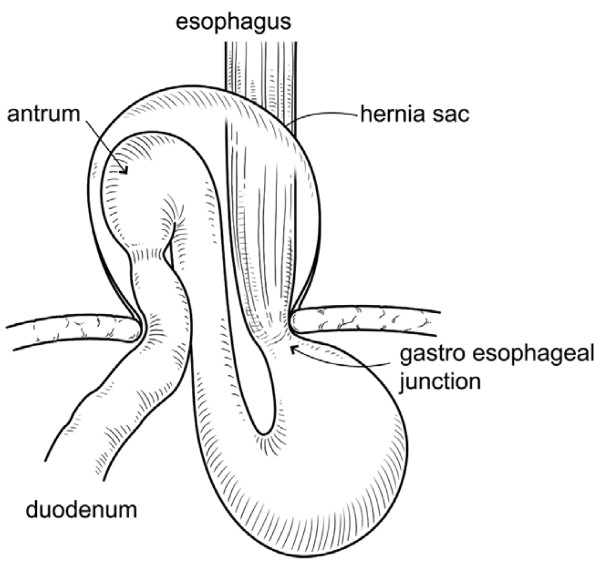
**Operative findings.** The prolapse of the gastric antrum and duodenal bulb into the mediastinum were revealed. The gastric antrum and duodenal bulb were associated with paraesophageal hernia.

The upper median laparotomic approach was used to expose the peritoneal cavity. The triangular ligament of the liver was incised to facilitate mobilization and rightward displacement of the left lobe of the liver. The gastric antrum and duodenal bulb involved in the paraesophageal hernia were noted on the right side of the esophagus (Figure 3). First, the gastric antrum was relocated to its normal position. Then the median arcuate ligament was exposed anterior to the right and left crura of the diaphragm and above the celiac artery. After exfoliating the tissue around the esophagus, the position of vagus nerve was confirmed. The median arcuate ligament was fixed to the anterior and posterior walls of the lesser curvature of the upper body of the stomach without damage to the vagus nerve. Finally, the right and left crura of the diaphragm were sutured using absorbable sutures, and the hiatal hernia was successfully corrected. The patient’s postoperative course was uneventful. On postoperative Day 7, plain abdominal computed tomography and gastrofluorography revealed a normal stomach, which was not dilated. The patient was ambulatory at discharge.

## Discussion

The incidence of esophageal hiatal hernia is increasing with the increase in the aging population, and approximately 60% of individuals aged > 50 years are affected with this condition [1]. Esophageal hernia is defined as prolapse of all layers of the stomach, including the serosa, into the mediastinum. It is most common among patients with diaphragmatic hernia. Prolapse of peritoneal organs other than the stomach into the mediastinum is rare.

Hiatal hernias are classified into 4 types [2]. In Type I, or sliding hiatal hernia, the gastroesophageal junction (GEJ) migrates cephalad through the hiatus into the thorax. In Type II, or paraesophageal hernia, the gastric fundus herniates through the hiatus into the thorax, but the GEJ remains in the abdomen. In Type III, which is a combination of types I and II, the GEJ and stomach herniate into the thorax. Type IV is a type III hiatal hernia with herniation of other organs into the thorax, such as the colon and spleen. Among these types of hernias, sliding hiatal hernia is the most common, accounting for 90%―95% cases of cases of esophageal hernia [3]. Paraesophageal hernia is the second most common and accounts for 5% of cases [4]. The etiologies of esophageal hiatal hernia include congenital opening of the esophageal hiatus, congenital deformity, acquired vulnerability of connective tissue because of obesity or aging, or increased abdominal pressure. In this case, the disorder was classified as paraesophageal hernia with involvement of the gastric antrum and duodenal bulb. Paraesophageal hernia presents most often in adults; therefore, acquired causes such as mechanical force and tissue degeneration are considered etiologic factors, although a congenital origin cannot be totally excluded.

The treatment for sliding esophageal hernia with mild gastroesophageal reflux is usually conservative. However, surgical treatment is recommended for sliding esophageal hernia refractory to conservative treatment, paraesophageal hernia liable to prolapse, or paraesophageal hernia with ulceration and/or stenosis. In cases of paraesophageal hernia, prolapse may suddenly occur, causing complications such as gastrointestinal necrosis by strangulation, gastric perforation, or massive hemorrhage. A high mortality rate is associated with paraesophageal hernia with complications; therefore, surgical treatment for paraesophageal hernia with or without complications is recommended.

Reports on the surgical treatment of esophageal hernia describe the repositioning of the hernial contents and closure of the hernial orifice. Two approaches are commonly used for the repair of esophageal hernias: the transthoracic approach and the transabdominal approach. The Allison [5] and Belsey― Mark IV technique [6] are transthoracic approaches. The techniques of Hill [7], Nissen [8], and Toupet [9] are transabdominal approaches. In this case, the transabdominal technique of Hill was used for inspection of the abdominal cavity, and, after repositioning the contents of the hernial sac, the superficial and deep layers of the diaphragmatic crura were easily closed using absorbable sutures. However, resection of the hernial sac could not be performed because of the transabdominal approach adopted in this case, but reportedly, it is not always necessary to perform the resection of the hernial sac [10].

A total of 9 case reports of esophageal hiatal hernia with incarceration of the gastric antrum and duodenal bulb were published between 2000 and 2013 (Table 1) [11-19]. In these reports, the male to female ratio of patients with this condition is 1:4, and the age at which diagnosis is made ranges from 52―88 years. The average BMI reported was 21.4 kg/m^2^. Therefore, the esophageal hiatal hernia with incarceration of the gastric antrum and duodenal bulb is not necessarily associated with obesity. Characteristic symptoms included vomiting (80%) and abdominal distension (20%). Almost all cases (90%) were treated surgically (laparotomy was used in 2 cases). Conservative treatment was successful in only 1 case. In 3 cases, duodenal perforations and incarceration of the gastric antrum and duodenal bulbs were detected at the time of diagnosis [12,13,16]. Esophageal hernia, whether of the sliding or paraesophageal type, can cause upper gastrointestinal perforation, including duodenal perforation; therefore, regular follow-up of patients with esophageal hernia is necessary. In particular, paraesophageal hernia with incarceration of other gastrointestinal organs is associated with a higher risk of upper gastrointestinal perforation. Therefore, surgical treatment should be considered in these cases.

**Table 1:** Cases of esophageal hiatal hernia with incarceration of the gastric antrum and duodenal bulb

**Reference**	**Year**	**Age**	**Sex**	**BMI**	**Symptom**	**Type of hernia**	**Therapy**
Eda [11]	2000	79	F	20.0	Vomiting	Combined	Surgery
Maruyama [12]	2001	71	F	NA	Abdominal pain, abdominal distension	Paraesophagus	Surgery
Otsuka [13]	2002	77	F	NA	Vomiting	Sliding	Surgery
Yoshioka [14]	2005	82	F	18.7	Epigastralgia, vomiting	Combined	Laparotomy
Itano [15]	2005	52	F	24.5	Abdominal distension, vomiting, weight loss	Paraesophagus	Surgery
Ekelund [16]	2006	88	M	NA	Vomiting, belching	Paraesophagus	Surgery
Nishida [19]	2008	75	M	NA	Vomiting	Paraesophagus	Laparotomy
Monma [18]	2010	71	F	21.1	Anorexia, chest burning tarry, stool	Paraesophagus	Surgery
Shinoda [19]	2010	85	F	22.2	Vomiting	Combined	Conservative
Our case	2013	84	F	22.1	Vomiting	Paraesophagus	Surgery

## Conclusion

We encountered a rare case of paraesophageal hernia with incarceration of the gastric antrum and duodenal bulb, which was corrected with surgical treatment because other treatment options failed to work. Therefore, even when complications are present, surgical treatment for paraesophageal hernia should be considered.

## Consent

Written informed consent was obtained from the patient for publication of this Case report and any accompanying images. A copy of the written consent is available for review by the Editor-in-Chief of this journal.

## Competing interests

The authors declare that they have no competing interests.

## Authors’ contributions

Wrote the first draft of the manuscript: NT, YN. Contributed to the writing of the manuscript: NT, YN. Agree with manuscript results and conclusions: NT, YN. Jointly developed the structure and arguments for the paper: NT, YN. Made critical revisions and approved final version: NT, YN. Both authors reviewed and approved of the final manuscript.
